# Clinical Rel mutations that increase basal (p)ppGpp promote conjugal transfer of staphylococcal resistance plasmids

**DOI:** 10.1099/mic.0.001724

**Published:** 2026-06-09

**Authors:** Ashley T. Deventer, Ava Sutherland, Daria Biernacka, Paul R. Johnston, Claire E. Stevens, Anna-Karina Kaczorowska, Alisdair B. Boraston, Joanne K. Hobbs

**Affiliations:** 1 Biomedical Sciences Research Complex, School of Biology, University of St Andrews, St Andrews, UK; 2 Faculty of Biology, Collection of Plasmids and Microorganisms, University of Gdańsk, Gdańsk, Poland; 3 School of Medicine, University of St Andrews, St Andrews, UK; 4 Department of Biochemistry and Microbiology, University of Victoria, Victoria, Canada

**Keywords:** conjugation, plasmid, (p)ppGpp, *Staphylococcus aureus*, stringent response

## Abstract

Conjugative transfer of plasmids represents a major route through which antibiotic resistance genes are spread. In the case of the prevalent and deadly pathogen *Staphylococcus aureus*, more than 90% of clinical isolates carry at least one plasmid. While plasmid-encoded mechanisms [e.g. plasmid copy number (PCN)] can influence conjugation frequency, host factors and environmental stimuli can also affect transmission. In particular, stress responses like the stringent response have been associated with increased movement of mobile genetic elements. We have previously shown that clinical mutations in the stringent response controller, Rel, lead to elevated levels of the alarmones guanosine tetra- and pentaphosphate [(p)ppGpp] and antibiotic tolerance in *S. aureus*. Here, we report that elevated (p)ppGpp in these strains promotes the conjugal transfer of diverse staphylococcal resistance plasmids. We observed that clinical Rel mutations promote donation, but not receipt, of plasmids from the three families of staphylococcal plasmid and a mobilizable plasmid. This increased conjugation frequency could also be induced by chemical induction of the stringent response by mupirocin. Intriguingly, detailed experimental analysis revealed that the effect of elevated (p)ppGpp on plasmid donation was not due to CodY derepression, SOS response induction or increased PCN. Furthermore, comparative transcriptomics of wild-type and mutant donor did not highlight any putative plasmid- or host-derived mechanisms to explain this observation. Further investigations are required to explore the mechanistic link between (p)ppGpp and conjugation, given the pervasive transcriptional and post-translational effects of (p)ppGpp. Overall, the association between Rel mutation and increased plasmid donation is alarming, especially as Rel mutations are being increasingly identified among clinical isolates.

## Data availability

Raw RNA sequencing data have been deposited under BioProject accession number PRJNA1349606: Newman (pGO1) – SAMN52925640-SAMN52925643; and Newman *rel* F128Y (pGO1) – SAMN52925636-SAMN52925639.

## Introduction

Horizontal gene transfer and mobile genetic elements (MGEs), like plasmids, are the main drivers behind the spread of antibiotic resistance [1,3]. Bacterial conjugation describes the process whereby genetic material is passed between two cells via direct contact. Conjugative plasmids are extra-chromosomal DNA elements that encode for all of the machinery necessary for their transfer, including mating pore proteins and a suite of other proteins required for DNA processing, replication and recruitment [4]. These plasmids also frequently encode multiple antibiotic resistance genes [5]. Conjugative plasmids are also capable of mobilizing non-conjugative plasmids when they reside in the same cell; estimates suggest that at least 80% of non-conjugative plasmids found in *Staphylococcus aureus* can be mobilized [4]. Therefore, conjugation is an important driver in resistance gene dissemination and contributor to the growing problem of antibiotic resistance globally.

*S. aureus* is a leading cause of many life-threatening infections and more than 90% of clinical isolates carry at least one plasmid [6]. These plasmids carry a huge diversity of resistance genes and many encode for resistance to multiple classes of antibiotic [7]. Conjugative plasmid transfer in *S. aureus* is initiated when a plasmid-encoded relaxase recognizes, cleaves and attaches to the origin of transfer located on the plasmid. This nucleoprotein complex (known as the relaxasome) then directs the plasmid DNA to the mating pore, where it is transferred into the recipient cell through a type-IV secretion system [4]. Mobilizable plasmids encode for all or part of the relaxasome but lack genes encoding for the mating pore. Therefore, they are dependent on a co-resident conjugative plasmid carrying compatible mating pore genes. Conjugative staphylococcal plasmids fall into three distinct families: the pSK41/pGO1 family, the pWBG749 family and the pWBG4 family [4]. These families differ in their conjugation gene clusters. The pSK41/pGO1 family is by far the best studied and understood [8]. pSK41 and pGO1 encode resistance to aminoglycosides, bleomycin and quaternary ammonium compounds. The two plasmids are essentially identical, but pGO1 contains a ~8 kb co-integrated plasmid (encoding trimethoprim resistance) [9]. The transfer region of pSK41/pGO1 contains most of the genes associated with conjugative plasmid transfer [8], while genes involved in replication, multimer resolution and plasmid partitioning are encoded in a second region, known as Region 1 [10]. The conjugation frequency (i.e. the rate of transfer) of a given plasmid is controlled by two factors: plasmid copy number (PCN) and the expression level of the conjugation machinery genes (*trs,* or transfer, genes) [11,12]. Logically, increased PCN and/or increased expression of transfer genes results in higher rates of plasmid transfer [13]. The PCN of pSK41/pGO1 is controlled by the replication initiation protein, Rep [14]. Because high rates of plasmid replication come with fitness costs for the host [15], expression of Rep is tightly controlled. The replication region of pSK41/pGO1, as well as encoding for Rep itself, encodes for two independent methods of Rep expression regulation. First, expression of Rep is transcriptionally, but predominantly translationally, controlled by an antisense RNA (RNAI) that is complementary to the *rep* mRNA leader region [14,16, 17]. Second, the replication region also encodes for a small transcriptional repressor protein, Cop, that binds to the *rep* promoter and represses *rep* transcription [18]. Expression of the transfer genes on pSK41/pGO1 is controlled by TrsN (known as ArtA in pSK41), a transcriptional repressor that binds to the promoters of *trsA, trsL* and three other promoters found within Region 1 [19,20].

While conjugation frequency can be increased or decreased by plasmid mutations [11,17], chromosomal mutations in the donor and environmental stimuli can also influence transmission rate [21,22]. In particular, stressors and the subsequently induced stress responses can promote increased conjugation frequency and mobilization of MGEs more generally [23,26]. Furthermore, conjugation is considered a stressful process for the donor and the induction of stress responses during mating is considered beneficial [27,28]. Induction of the SOS response has long been known to promote transmission of MGEs in both Gram-negatives and Gram-positives [25,26]. More specifically, SOS response induction by antibiotics and other pharmaceuticals leads to increased conjugation rates [22,24, 29, 30] despite reducing the rate of plasmid replication [31]. A second bacterial stress response – the stringent response – has also been implicated in elevated MGE transmission. Strugeon *et al*. observed that *Escherichia coli* growing in a biofilm had a higher rate of MGE excision than planktonic cells due to induction of the stringent response [23]. Biofilms are often considered ‘hotspots’ for horizontal gene transfer [32] and the stringent response is frequently implicated in biofilm formation [33]. This stress response has also been postulated to play a role in MGE transfer in the plant symbiote *Mesorhizobium japonicum* [34]. The stringent response is a conserved stress response mediated by the ‘alarmones’ guanosine tetra- and pentaphosphate [collectively known as (p)ppGpp] (reviewed in [35]). In most bacteria, cellular levels of (p)ppGpp are mediated by a bifunctional enzyme, Rel, that both synthesizes and hydrolyses (p)ppGpp via two distinct catalytic domains. Some bacteria, such as *S. aureus,* also possess accessory (p)ppGpp synthetases. In the classical description of the stringent response, limitation of one or more amino acids results in uncharged tRNAs entering the ribosome, resulting in stalling. This stalling is sensed by Rel, which responds by synthesizing (p)ppGpp from ATP and GDP/GTP. The downstream effectors of (p)ppGpp vary greatly between species, with >50 known binding partners of (p)ppGpp [36]. However, the overall effect of elevated (p)ppGpp is a downregulation of most metabolic processes and derepression of genes associated with amino acid synthesis and import. In *S. aureus*, the derepression effects of (p)ppGpp are mediated via CodY, a transcriptional repressor that requires GTP in order to bind to DNA [37]. While the stringent response was once thought to be binary, either ‘on’ or ‘off’, we now know that a basal level of (p)ppGpp is present in all cells, and (p)ppGpp acts as a master regulator of almost all aspects of bacterial physiology and virulence [38].

Given the pervasive cellular effects of (p)ppGpp, it is not surprising that it has been found to play a role in various aspects of antibiotic susceptibility, resistance and killing [39,41]. We have previously observed that Rel mutations identified in clinical isolates confer antibiotic tolerance in *S. aureus* by elevating basal (p)ppGpp and slowing growth of the bacterium [42,43]. Antibiotic tolerance describes the ability of a bacterial population to survive transient exposure to an otherwise lethal concentration of antibiotic without exhibiting resistance [44]. It is emerging as an important contributor to persistent, relapsing and recurrent infections, as well as, worryingly, acting as a precursor to the development of endogenous resistance [40,45]. Given that plasmid conjugation is a major contributor to the spread of antibiotic resistance, and stress responses have been implicated in the transmission of MGEs, we set out to investigate whether clinical Rel mutations and elevated (p)ppGpp impact plasmid transmission rates in *S. aureus*. Here, we show that strains bearing Rel mutations donate plasmids at significantly higher frequencies than wild-type strains. This effect applies to the transfer of diverse conjugative and mobilizable plasmids, but intriguingly, the molecular basis of this effect is neither increased PCN nor expression of transfer genes. As Rel mutations are now being identified among further clinical isolates [46,48], this emerging relationship with plasmid transfer requires further investigation.

## Methods

### Reagents and antibiotics

X-gal (5-bromo-4-chloro-3-indolyl-β-d-galactopyranoside) and chloramphenicol were purchased from Bio Basic Inc. (Markham, ON, Canada). All other antibiotics and reagents, unless otherwise stated, were purchased from MilliporeSigma/Merck.

### Bacterial strains, plasmids and growth conditions

All bacterial strains and plasmids used in this study are listed in Table S1, available in the online version of this article. *S. aureus* strains were routinely cultured in tryptic soy broth (TSB) and on tryptic soy agar (TSA) at 37 °C, while *E. coli* strains were cultured in/on Lysogeny Broth (LB) or agar. The Newman *rel* mutants F128Y and L152F and their complemented counterparts (F128Y comp and L152F comp) have been reported previously [42], as has the *rel* F128Y mutant of USA300 LAC [43]. Strains were made novobiocin-resistant (NOVR) as previously described [43]; all strains were confirmed (via PCR and sequencing) to carry the same *gyrB* R144I mutation. SH1000 was made novobiocin- and rifampicin-resistant by plating on TSA containing 1 µg ml^−1^ novobiocin and 0.5 µg ml^−1^ rifampicin, while Newman F128Y comp was made resistant to fusidic acid by plating on TSA containing 1 µg ml^−1^ fusidic acid.

### Mutant generation via allelic exchange

To delete residues 308–310 from Rel (GenBank accession number NWMN_RS08620) and inactivate the synthetase domain, the two flanking halves of the gene were amplified from Newman genomic DNA and joined together by overlap extension PCR. For the *relP* and *relQ* knockouts, left and right flanks of each gene (and surrounding genes when needed to give fragments >500 bp) were amplified and joined together, excluding an internal fragment that contains conserved domains responsible for synthetic activity [49]. For *codY*, a complete deletion of the gene (except the start and stop codons) was generated by amplifying the up- and downstream genes (and intergenic regions) and joining together. The *lexA* S130A insert was generated by amplifying at least 500 bp up- and downstream of the mutation site with mutagenic primers and joining together. All inserts were cloned into pIMAY-Z [50] between the EcoRI and NotI sites via In-Fusion cloning (TaKaRa Bio), transformed into *E. coli* Stellar, and plated on LB agar containing 10 µg ml^−1^ chloramphenicol and 50 µg ml^−1^ X-gal at 37 °C. Confirmed pIMAY-Z constructs were transformed into *E. coli* IM08B [50], extracted, electroporated into competent *S. aureus* cells and integrated/excised as previously described [42]. Mutants were confirmed initially by PCR and bidirectional sequencing and later by long-read whole genome sequencing (Novogene). All primers are listed in Table S2. For construction of Δ*rel_syn_* Δ*relP*Δ*relQ*, the *rel*_syn_ mutation was introduced first, followed by the *relP* deletion and then the *relQ* deletion.

### Introduction of plasmids into donor strains via conjugation

Conjugative plasmids were introduced into the different NOVR donor strains via conjugation with NOV-sensitive hosts. Unmarked SH1000 (pGO1) was used to conjugate pGO1, while WBG541 [51] was used as the source of pWBG749e [52]. pWBG707 [53] was provided in a NOVR strain, WBG4515 [54], so pWBG707 was first conjugated into Newman F128Y comp FUSR before being transferred into the NOVR donor strains. To introduce both pGO1 and pC221 [55] into NOVR donor strains, pGO1 was first conjugated into RN4220 (pC221) and the transconjugant was then used to introduce both plasmids into the NOVR strains simultaneously. These conjugations were performed essentially as described below, except matings were incubated for 24 h and no quantitation of donor and recipient was performed. Transconjugants were restreaked on antibiotic plates to ensure purity. Antibiotic concentrations used to select for plasmids and chromosomal resistance were: gentamicin (5 µg ml^−1^); trimethoprim (10 µg ml^−1^); erythromycin (5 µg ml^−1^); chloramphenicol (10 µg ml^−1^); novobiocin (1 µg ml^−1^); rifampicin (5 µg ml^−1^) and streptomycin (50 µg ml^−1^).

### Conjugation/mobilization frequency determinations

**Fig. 1.**
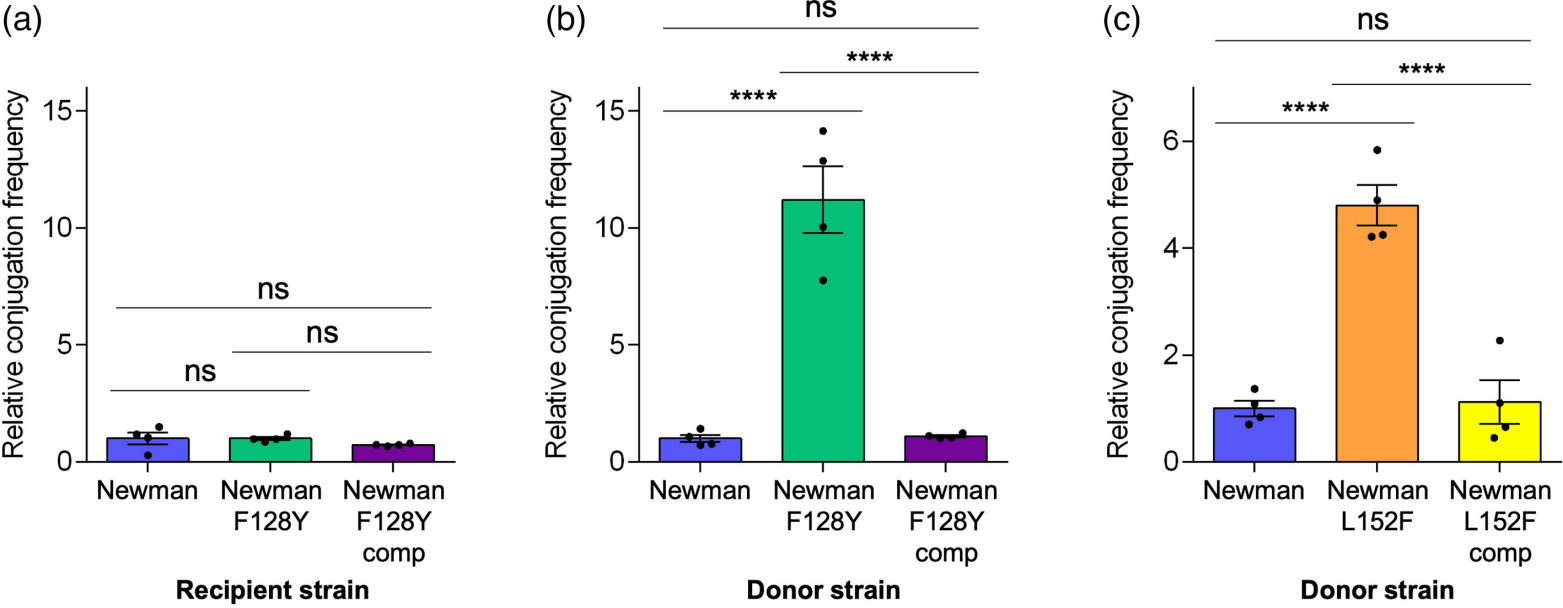
Clinical Rel mutations increase the frequency of pGO1 donation, but not receipt. (**a**) Filter matings were performed with SH1000 (pGO1) as donor and the three NOV^R^ recipients indicated. (**b**) Filter matings were performed with NOV^R^ wild-type Newman, Newman bearing the *rel* F128Y mutation and a complemented version of the F128Y mutant where the mutation has been reversed on the genome as donors (each carrying pGO1) and SH1000-NR as the recipient. (c) Filter matings were performed with NOV^R^ wild-type Newman, Newman bearing the *rel* L152F mutation and a complemented version of the L152F mutant where the mutation has been reversed on the genome as donors (each carrying pGO1) and SH1000-NR as the recipient. All conjugation frequencies were calculated as transconjugants per donor and are expressed relative to the Newman mean. Data shown are the mean of four biological replicates; error bars represent the sem. Asterisks indicate statistically significant differences between means as determined by a one-way ANOVA with Tukey’s multiple comparisons test (****P≤0.0001; ns=not significant).

Conjugation and mobilization frequencies were determined as described by Savage *et al*. [56] with some modifications. Donor and recipient strains were grown on selective agar at 37 °C overnight then resuspended in antibiotic-free TSB to an OD_600 nm_ of 1.0. Where chemical stringent response induction was required, donors were streaked out on TSA containing a range of sub-inhibitory concentrations of mupirocin and the concentration that permitted growth but induced a small colony phenotype was used (0.04 µg ml^−1^ for Newman and LAC). When acting as the donor, wild-type and complemented strains were combined with the recipient at a 1 : 2 ratio (250 µl wild-type/complemented and 500 µl SH1000-NR), while mutant strains were combined with SH1000-NR at a 1 : 1 ratio (500 µl each). This was found necessary to ensure a consistent donor-to-recipient ratio between strains (a parameter that can impact conjugation frequency [57]). Mating mixtures were pushed through a syringe onto a 0.2 µM pore-size nylon filter using 13 mm Swinnex filter holders. Filters were placed bacteria side down on TSA and incubated at 37 °C for 4 h. Viable counting of mating mixtures immediately after mixing and post-incubation revealed little growth of the donor and recipient strains during this time. Following incubation, cells from filters awere resuspended in 1 ml TSB and serially diluted in PBS. Serial dilutions were plated on TSA containing the appropriate antibiotics for selection of donors, recipients or transconjugants and incubated at 37 °C for 16–24 h. For the matings shown in Fig. 1a, the donor was selected for and enumerated by plating on gentamicin, recipients were enumerated by plating on novobiocin and transconjugants were enumerated by plating on novobiocin + gentamicin. In all other matings, donors were enumerated by plating on novobiocin plus the selective agent(s) for the specific plasmid(s) (gentamicin for pGO1, trimethoprim for pWBG707, erythromycin for pWG749e and chloramphenicol for pC221); recipients were enumerated by plating on novobiocin + rifampicin; and transconjugants were enumerated by plating on novobiocin + rifampicin plus the selective agent(s) for the specific plasmid(s). Each dilution was plated out in duplicate and only plates with between 30 and 300 colonies were counted. Colonies were counted either manually or using an aCOLyte 3 automated colony counter (Synbiosis Ltd., Cambridge, UK). Four independent matings were performed per donor-recipient combination/condition. The donor-to-recipient ratio was calculated for each mating and any matings where the ratio fell below 0.5 : 1 or above 1.5 : 1 were excluded. Conjugation frequencies were calculated as the number of transconjugants (in c.f.u./ml) per donor [56]. Mobilization frequencies were calculated as the number of pC221-containing transconjugants per donor [56].

### Antibiotic susceptibility assays

Minimum inhibitory concentrations (MICs) were determined in triplicate in Mueller-Hinton broth following the broth microdilution guidelines of the Clinical and Laboratory Standards Institute (CLSI) [58]. Population analysis profiles with gentamicin were determined by growing the test strains overnight in TSB + 5 µg ml^−1^ gentamicin, diluting in phosphate-buffered saline and plating on TSA containing increasing concentrations of gentamicin. Plates were incubated for 24 h prior to colony counting. Three independent overnight cultures per strain were plated.

### Reporter construct generation

The staphylococcal reporter vector pJB185 [59] was amplified and linearized by PCR using primers listed in Table S2, and the methylated template DNA degraded with DpnI. The pGO1 *rep* promoter region (including RNAI) and the *trsA* promoter were amplified from a lysed colony of SH1000 (pGO1). Inserts were cloned into pJB185 via In-Fusion cloning, transformed into *E. coli* Stellar and plated on ampicillin. Following confirmation of insert by bidirectional sequencing, purified plasmids were transformed into *E. coli* IM08B, extracted and introduced into *S. aureus* strains either by electroporation or phage transduction with phi85 [60].

### Chloramphenicol acetyltransferase reporter assay

The PCN reporter construct pSK5487 was introduced into Newman strains via phage transduction. For the reporter assay, the method of Kwong *et al*. [14] was followed with a few modifications. Briefly, samples of overnight cultures grown in TSB were pelleted, resuspended in lysis buffer containing 0.13 mg ml^−1^ lysostaphin and incubated at 37 °C for 30 min prior to sonication. Insoluble material was then pelleted and the supernatants diluted 1/10 prior to use. For the chloramphenicol acetyltransferase (CAT) assay, 93 µl CAT assay mixture (100 mM Tris, pH 7.8, 0.1 mM acetyl-CoA, 1 mM 5,5′-dithiobis-(2-nitrobenzoic acid) and 5 µl diluted cell supernatant were combined in a 96-well plate and equilibrated to 37 °C for 2 min in a Tecan Spark plate reader. The reaction was initiated with 2 µl 5 mM chloramphenicol, shaken for 5 s and the absorbance at 415 nm monitored. The initial rate of CAT activity was determined from the slope and converted to μM min^−1^ using the extinction coefficient of the product 5-thio-2-nitrobenzoic acid (14,150 M cm^−1^) and a pathlength of 0.28 cm. The total protein content of each cell supernatant was determined using a Bradford assay and this value used to normalize the rate of CAT activity. Three independent cultures were grown, lysed and assayed per strain.

### LacZ reporter assays

The *rep* and *trsA* reporter constructs pJB185-*rep* and pJB185-*trsA* were introduced into Newman strains via phage transduction. Cell extracts were prepared as described for the CAT report assay, except that cells from mid-exponential phase were pelleted and lysed. The Beta-Glo^®^ assay system (Promega) was used to quantify LacZ expression in 50 µl diluted cell supernatants following the manufacturer’s instructions. Total protein content of each cell supernatant was determined as described above and used to normalize the luminescence values. Three independent cultures were grown, lysed and assayed per strain.

### Droplet digital PCR

Droplet digital PCR (ddPCR) was used for the absolute quantification of pGO1 PCN in Newman wild-type, F128Y and F128Y comp. For these assays, triplicate 2 ml overnight cultures of each strain grown in TSB (supplemented with gentamicin) were harvested by centrifugation, cells washed twice with PBS and resuspended in 1 ml of PBS. The suspension was subjected to bead-beating using the standard B-matrix protocol and a FASTPREP-24 5G instrument (MP Biomedicals, USA). The homogenate was centrifuged for 5 min at 13,000 r.p.m., and the supernatant containing total DNA retained. One microlitre of a 10⁻⁴ dilution in sterile water was used as the template for ddPCR. Each ddPCR reaction (25 µl total volume) contained nuclease-free water, 2× EvaGreen ddPCR Supermix (Bio-Rad) and primers at a final concentration of 0.1 µM. Primer sequences for amplification of *femA* (chromosomal; NWMN_RS07245) [61] and *aphD* (pGO1 plasmid; PGO1_RS00250) fragments are provided in Table S2. No-template controls were included to monitor contamination and primer–dimer formation. Reactions were dispensed in duplicate into a 96-well semi-skirted PCR plate. Plates were heat-sealed with pierceable foil, vortexed briefly, centrifuged for 1 min and loaded into an AutoDG instrument (Bio-Rad) for droplet generation using EvaGreen AutoDG oil. The resulting droplet plate was heat-sealed and transferred to a C1000 Touch Thermal Cycler (Bio-Rad). PCR amplification was performed according to the manufacturer’s instructions under the following cycling conditions: 95 °C for 2 min (1 cycle); 30 cycles of 94 °C for 30 s (ramp rate 2 °C min^−1^) and 50 °C for 30 s (ramp rate 2 °C min^−1^), 72 °C for 2 min; followed by 70 °C for 10 min and a final hold at 8 °C until droplet reading. After amplification, the plate was loaded onto a QX200 Droplet Reader (Bio-Rad) and analysed using QuantaSoft software version 1.7.4 (Bio-Rad). Each droplet functions as an independent PCR reaction and fluorescence detection distinguishes positive from negative droplets. For each well, the concentration of target molecules was calculated based on the fraction of positive droplets assuming a Poisson distribution. The associated error for each well was reported as the 95% confidence interval derived from the Poisson distribution, reflecting the statistical uncertainty of target molecule partitioning into droplets. PCN was calculated as the ratio of (copies/µl plasmid gene) to (copies/µl chromosomal gene). PCN values represent the mean of two technical replicates derived from three independently prepared DNA samples per strain.

### RNA sequencing

Cells for RNA extraction were grown exactly as described for conjugation frequency determinations. Following the 4 h incubation, filters were removed from plates, the cells resuspended in 1 ml TSB and combined with two volumes of RNAprotect (Qiagen). From here, total RNA samples were prepared, quantified and sequenced by Novogene as described previously [62]. RNA was extracted from four independent filter matings per donor. Gene expression was quantified using Salmon v1.10.2 [63] by pseudoaligning reads to CDS from NC_009641.1 [64] and NC_012547 [9]. The resulting counts were modelled as a function of strain using DESeq2 1.40.2 [65]. Differential gene expression was determined by a threshold of absolute fold change greater than two and an FDR-adjusted *P*-value less than 0.05. Manual inspection of strand-specific aligned reads was performed in Artemis [66]. Gene ontology (GO) enrichment analysis was performed on Biological Process terms associated with differentially expressed genes using the GOstats R package [67]. Enriched terms were identified using a conditional hypergeometric test and significance was defined as an FDR-adjusted *P*-value<0.05.

## Results

### Rel mutation promotes pGO1 donation but not receipt

We have previously introduced two clinical Rel mutations – F128Y and L152F – into the *S. aureus* model strain Newman and shown that they partially activate the stringent response by shifting the activity of Rel in favour of (p)ppGpp synthesis [42,43]. Combined evidence from growth curves, (p)ppGpp quantitation and enzyme assays suggests that the F128Y mutation induces a greater induction of the stringent response than L152F [42,43]. In order to investigate the impact of these mutations on conjugal plasmid transfer, we began by measuring the conjugation frequency of pGO1 from SH1000 into NOV^R^ derivatives of wild-type Newman, Newman F128Y and a complemented strain (where the Rel F128Y mutation has been reversed). Because *S. aureus* conjugation requires cell-to-cell contact and growth in a biofilm [56], strains were combined and mated on nylon filters for 4 h prior to plating out for donor, recipient and transconjugant quantification. There was no significant difference in the frequency of plasmid uptake by Newman, F128Y or the complemented strain (Fig. 1a). However, when the matings were reversed and the Newman strains acted as pGO1 donors, the conjugation frequency for the F128Y mutant was ~tenfold higher than that of the wild-type and complemented strains (Fig. 1b). A similar, but smaller, effect was seen when the L152F mutant was used as the donor (Fig. 1c). We confirmed that this effect was not due to a difference in donor-to-recipient ratio between strains (a parameter that can impact conjugation frequency [57]); only data where the ratio fell between 0.5 : 1 and 1.5 : 1 were analysed, and collated data demonstrate that there is no correlation between conjugation frequency and donor-to-recipient ratio within this range (Fig. S1). During optimization of mating conditions and donor-to-recipient ratio, various volumes of donor and recipient cells were trialled (including fixed initial ratios) and an elevated conjugation frequency in Rel mutant donors was consistently observed. Therefore, our Rel mutants donate plasmids to other strains of *S. aureus* at a higher frequency than wild-type donor, but their ability to receive a plasmid via conjugation is unchanged.

### (p)ppGpp modulation affects the frequency of donation of diverse plasmids

**Fig. 2.**
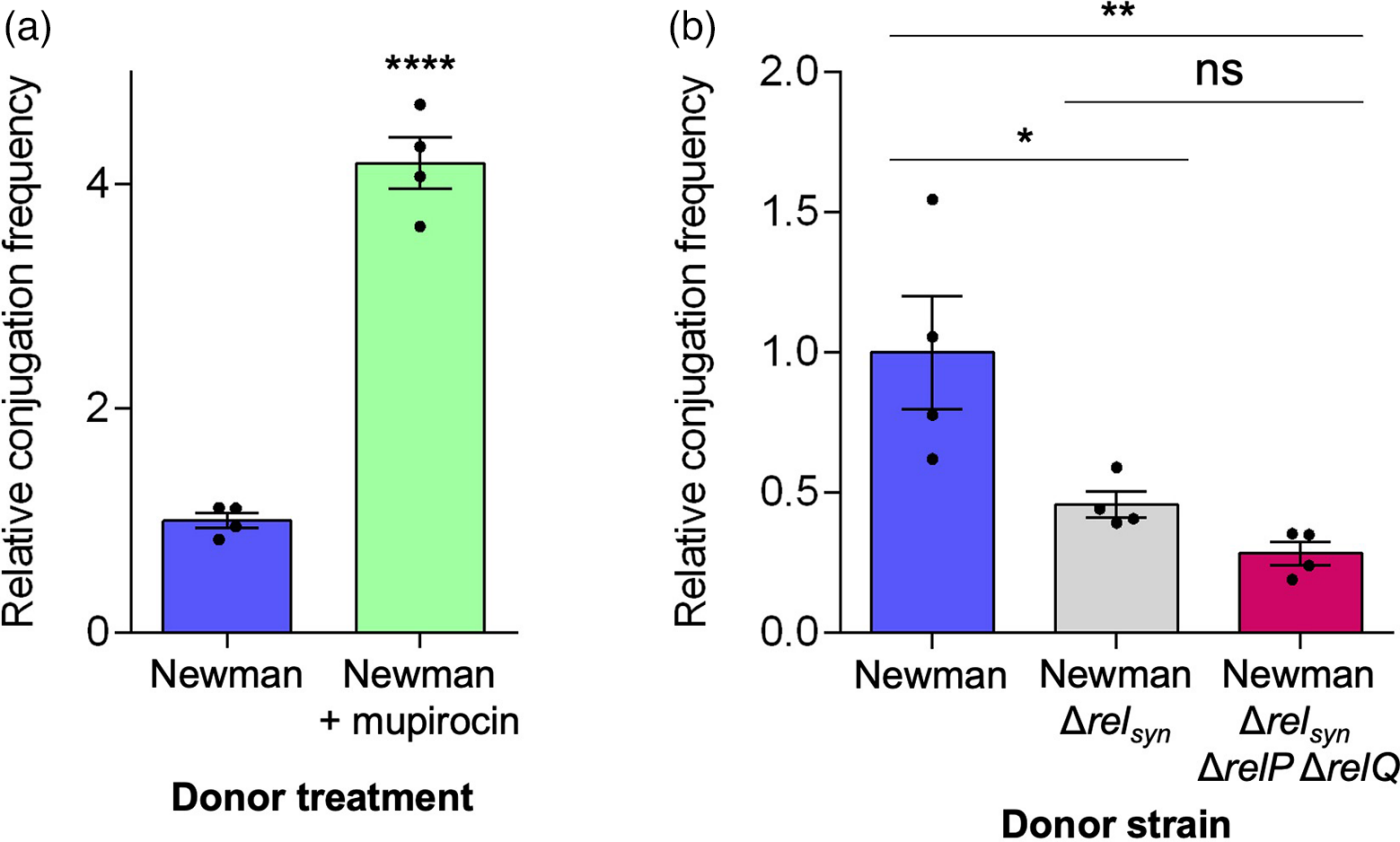
Modulation of (p)ppGpp level affects the frequency of pGO1 donation. Filter matings were performed with the NOV^R^ donor indicated and SH1000-NR as the recipient. (**a**) Conjugation frequency for transfer of pGO1 from Newman with and without chemical induction of the stringent response with mupirocin (donor was exposed to a subinhibitory concentration of mupirocin prior to mating). (**b**) Conjugation frequency for transfer of pGO1 from wild-type Newman and two stringent response-defective mutants. All conjugation frequencies were calculated as transconjugants per donor and are expressed relative to the Newman mean. Data shown are the mean of four biological replicates; error bars represent the sem. Asterisks indicate statistically significant differences between means as determined by a two-tailed t-test in panel A and a one-way ANOVA with Tukey’s multiple comparisons test in panel B (*P≤0.05; **P≤0.01; ****P≤0.0001; ns=not significant).

In addition to mutation of Rel, the stringent response can be activated in bacteria by exposure to mupirocin, an isoleucyl-tRNA synthetase inhibitor. To corroborate our results with the Rel mutants, we exposed wild-type Newman carrying pGO1 to a subinhibitory concentration of mupirocin overnight prior to mating. Exposure to mupirocin induced a ~fourfold increase in conjugation frequency compared with the unexposed control (Fig. 2a). This effect of mupirocin, and the effect of the Rel F128Y mutation, on conjugation frequency is not strain-specific, as we observed the same trend with USA300 LAC (Fig. S2). In *S. aureus*, while (p)ppGpp is primarily synthesized by Rel, there are two accessory (p)ppGpp synthetases that can contribute (RelP and RelQ). To test whether decreasing the cellular (p)ppGpp concentration would reduce plasmid donation, we generated a Δ*rel*_syn_ mutant of Newman that retains (p)ppGpp hydrolysis activity but lacks synthetic activity (Table S1) [68]. In this Δ*rel*_syn_ background, we also deleted *relP* and *relQ* to generate a (p)ppGpp-null strain [49]. Deletion of Rel synthetase activity led to a small (~twofold) but significant decrease in conjugation frequency compared with wild-type Newman (Fig. 2b). When the (p)ppGpp-null mutant was used as the donor, the conjugation frequency was decreased slightly further. However, plasmid transmission still occurred, indicating that while elevated (p)ppGpp can promote conjugal plasmid transfer, it is not essential for conjugation.

**Fig. 3.**
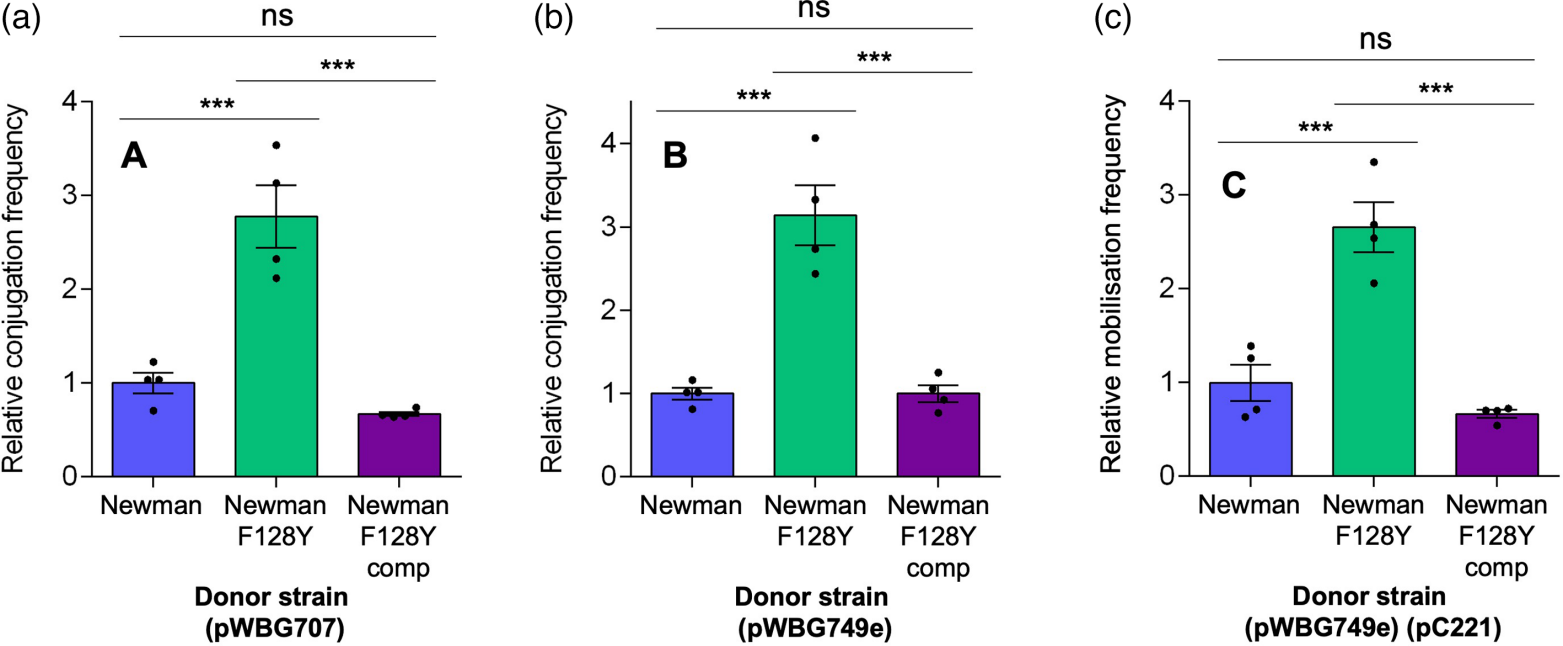
Clinical Rel mutation increases the donation frequency of diverse staphylococcal conjugative and mobilizable plasmids. Filter matings were performed with different NOV^R^ donors (as indicated) carrying different staphylococcal plasmids and SH1000-NR as the recipient. (**a**) Conjugation frequency for transfer of pWBG707 from different Newman strains. (**b**) Conjugation frequency for transfer of pWBG749e from different Newman strains. (**c**) Mobilization frequency for transfer of pC221 by pWBG749e from different Newman strains. All conjugation/mobilization frequencies were calculated as transconjugants per donor and are expressed relative to the Newman mean. Data shown are the mean of four biological replicates; error bars represent the sem. Asterisks indicate statistically significant differences between means as determined by a one-way ANOVA with Tukey’s multiple comparisons test (***P≤0.001; ns=not significant).

Next, we decided to test whether the effect of elevated (p)ppGpp on pGO1 conjugation frequency would also apply to other plasmids. pWBG707 and pWBG749e are representative conjugative resistance plasmids from the pWBG4 and pWBG749 families, respectively. They differ from pGO1 in their size (Table S1), resistances and conjugation genes [4]. Conjugation of pWBG707 and pWBG749e from the F128Y mutant into SH1000 occurred at significantly higher frequencies than when Newman wild-type or the complemented strain were used as donors (although the effect was not as pronounced as with pGO1; Fig. 3a, b). The F128Y mutant also mobilized pC221 when it was co-resident in cells with pWBG749e at a higher frequency than the wild-type and complement (Fig. 3c). Therefore, the positive effect of Rel mutation on plasmid donation appears to be universal and not specific to the pSK41/pGO1 plasmid family.

### (p)ppGpp does not promote plasmid donation via CodY derepression, induction of the SOS response or increased plasmid copy number

**Fig. 4.**
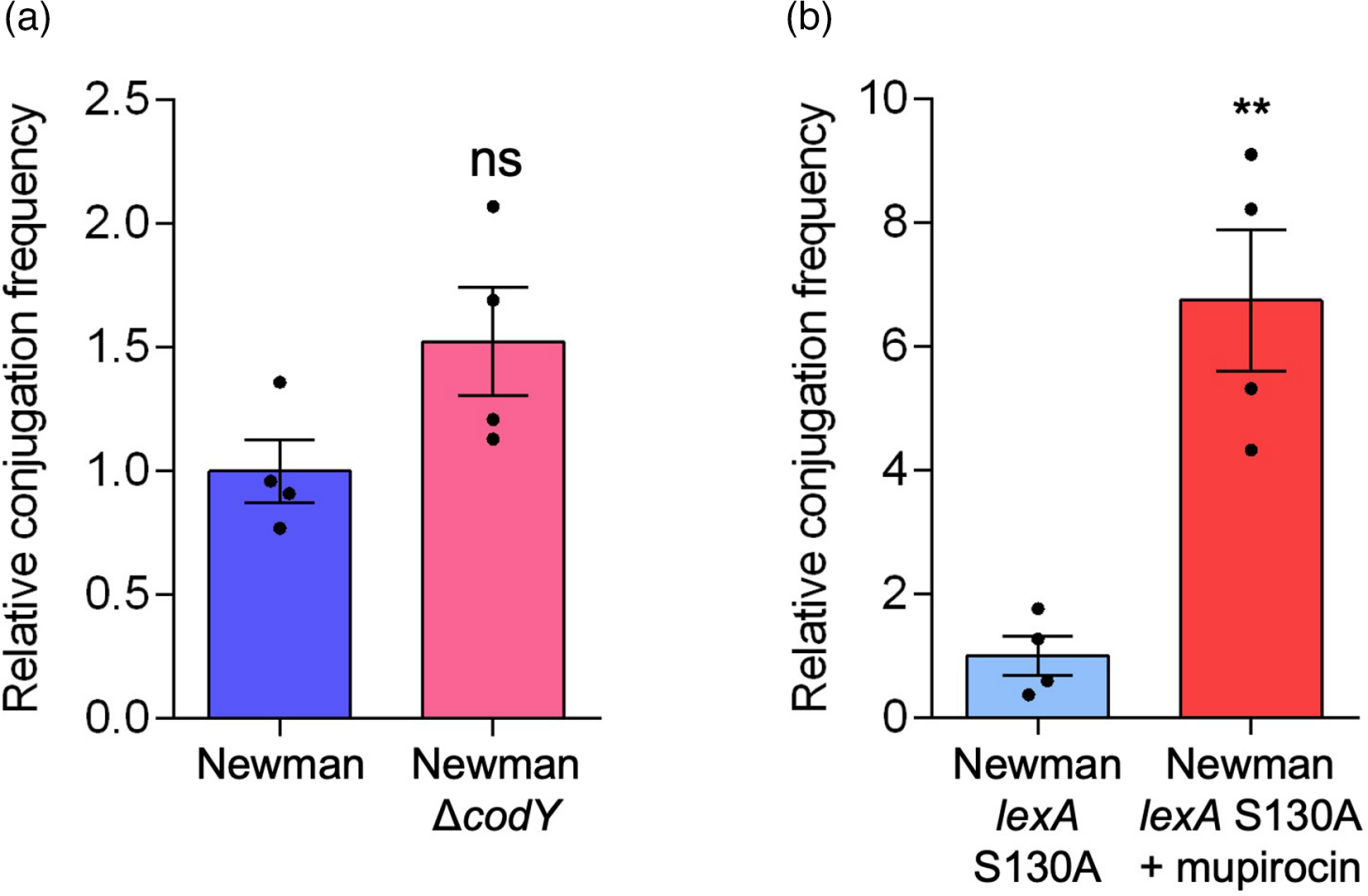
(p)ppGpp does not promote donation of pGO1 via CodY derepression or induction of the SOS response. Filter matings were performed with the NOV^R^ donor indicated and SH1000-NR as the recipient. (**a**) Conjugation frequency for transfer of pGO1 from wild-type Newman and a Δ*codY* mutant. (**b**) Conjugation frequency for transfer of pGO1 from a *lexA* mutant with and without chemical induction of the stringent response with mupirocin (donor was exposed to a subinhibitory concentration of mupirocin prior to mating). All conjugation frequencies were calculated as transconjugants per donor and are expressed relative to the wild-type Newman or no mupirocin mean. Data shown are the mean of four biological replicates; error bars represent the sem. Asterisks indicate statistically significant differences between means as determined by a two-tailed t-test (**P≤0.01; ns=not significant).

Next, we were interested to investigate the molecular mechanism behind the (p)ppGpp and plasmid conjugation relationship. As mentioned previously, part of stringent response activation in *S. aureus* is release of the transcriptional repressor CodY from transcripts. To test whether CodY derepression may play a role in increased plasmid transfer by stringent response-activated donors, we compared the conjugation frequency of pGO1 between wild-type Newman and a *codY* deletion mutant as donors (Fig. 4a). The Δ*codY* strain exhibited a slightly higher conjugation frequency than the wild-type, but the difference was not statistically significant and not on the same scale as the F128Y mutant or mupirocin treatment. We also tested the potential contribution of the SOS response. The stringent and SOS responses are intrinsically linked [69] and a previous study found that increased MGE transmission upon stringent response induction was due to downstream activation of the SOS response [23]. The SOS response is mediated by the repressor LexA, which must undergo self-cleavage for the SOS response to be induced. Therefore, mutation of the catalytic serine (S130) in LexA prevents SOS induction [70]. We generated a LexA S130A mutant of Newman and exposed it to mupirocin to test whether a functional SOS response is required for (p)ppGpp-stimulated plasmid donation (Fig. 4b). The LexA mutant exhibited a large and significant increase in conjugation frequency following mupirocin exposure, indicating that the SOS response is not necessary for the relationship between (p)ppGpp and plasmid transfer.

Increased conjugation frequency can occur due to elevated PCN and PCN can be phenotypically reflected in the level of resistance of a strain [11]. Therefore, we compared the pGO1-encoded resistance of wild-type Newman, the F128Y mutant and the complemented strain. Interestingly, the wild-type and complemented strains had MICs for gentamicin of 50 and 75 µg ml^−1^, respectively, while the F128Y mutant had a reproducible MIC of 200 µg ml^−1^. The higher gentamicin resistance phenotype of F128Y compared with the wild-type was also confirmed by population analysis profile determination (Fig. S3). However, when the MIC for trimethoprim was determined, the F128Y mutant had an MIC of 1,400 µg ml^−1^ while the wild-type and complemented strains had MICs of ≥1,500 µg ml^−1^.

**Fig. 5.**
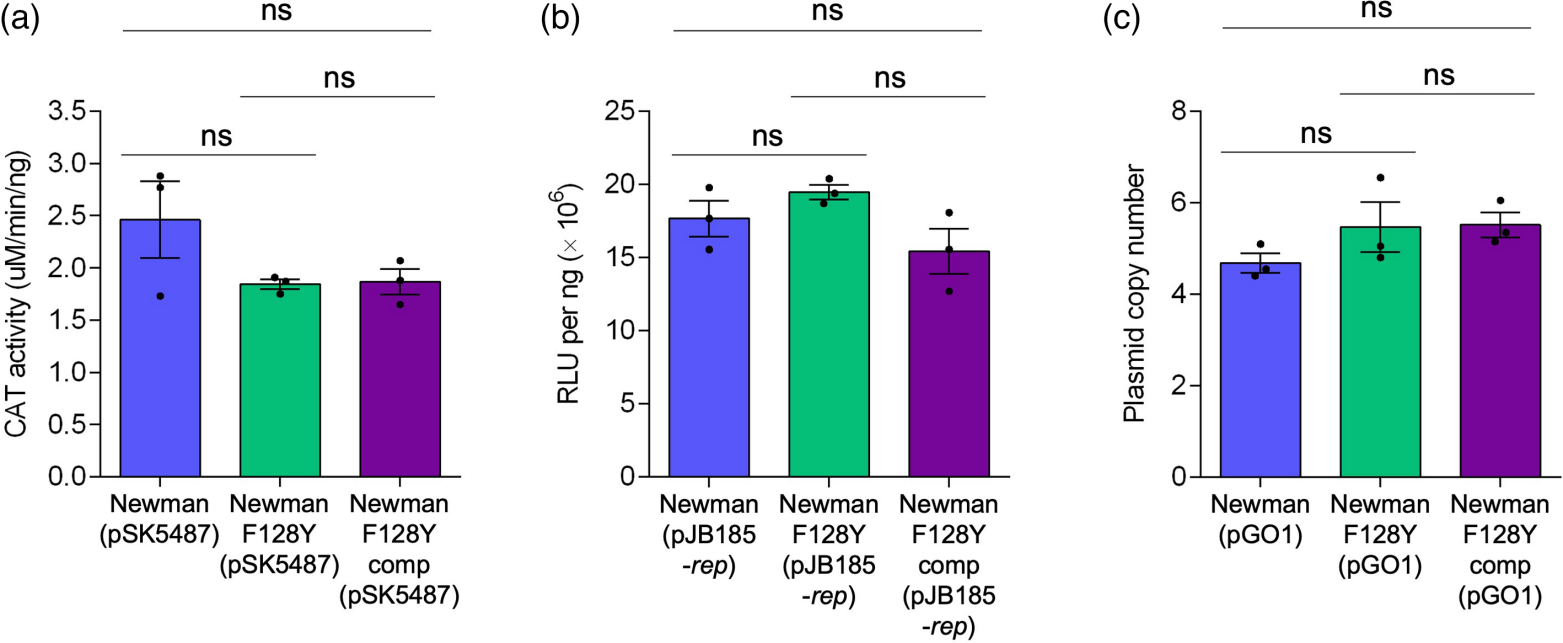
Clinical Rel mutation does not increase pGO1 copy number. (**a**) CAT activity in different strains carrying pSK5487, a PCN reporter construct bearing the pGO1 replication region. (**b**) LacZ activity in different strains carrying pJB185-*rep*, a reporter construct in which expression of *lacZ* is under the control of the pGO1 *rep* promoter region. In both panels, data were corrected for total protein content and are derived from three independent replicate cultures per strain (and two technical replicates). (**c**) Absolute PCN of pGO1 for each strain as determined by ddPCR. Data shown are the mean of three independent biological replicates and two technical replicates. In all panels, error bars represent the sem. Statistically significant differences between means were assessed by a one-way ANOVA with Tukey’s multiple comparisons test (ns=not significant).

To further explore whether PCN is elevated in the F128Y mutant, two reporter constructs were employed. First, a PCN reporter construct for pGO1/pSK41 has previously been reported [17]. pSK5487 is a mini replicon encoding for CAT and whose replication in *S. aureus* is dependent on the pGO1/pSK41 replication region. We introduced pSK5487 into wild-type Newman, F128Y and the complemented strain and quantified CAT activity in cell lysates. As elevated (p)ppGpp is associated with global downregulation of protein translation, we expressed the CAT activity as per ng of total protein. No significant difference in CAT activity was observed between the three strains (Fig. 5a). As the PCN of pGO1 is regulated by Rep and the antisense RNAI, we also constructed a reporter construct in which the expression of LacZ is under the control of the complete pGO1 *rep* promoter region and introduced this into our three strains. Again, we saw no significant difference in reporter enzyme activity between strains (Fig. 5b). Finally, to provide a measure of absolute PCN that would not be potentially confounded by the metabolic downregulation mediated by elevated (p)ppGpp, we performed ddPCR on DNA extracted from our three strains (Fig. 5c). The PCN of pGO1 in wild-type Newman was determined to be ~5, which is consistent with other PCN estimates for similarly large plasmids [71]. The PCN of pGO1 in F128Y was not statistically significantly different from that of wild-type Newman or the complemented strain. Therefore, we can conclude from this body of evidence that elevated PCN is not the molecular basis of increased conjugation frequency in our stringent response-activated strains.

### Plasmid and chromosomal transcriptome of F128Y (pGO1)

**Fig. 6.**
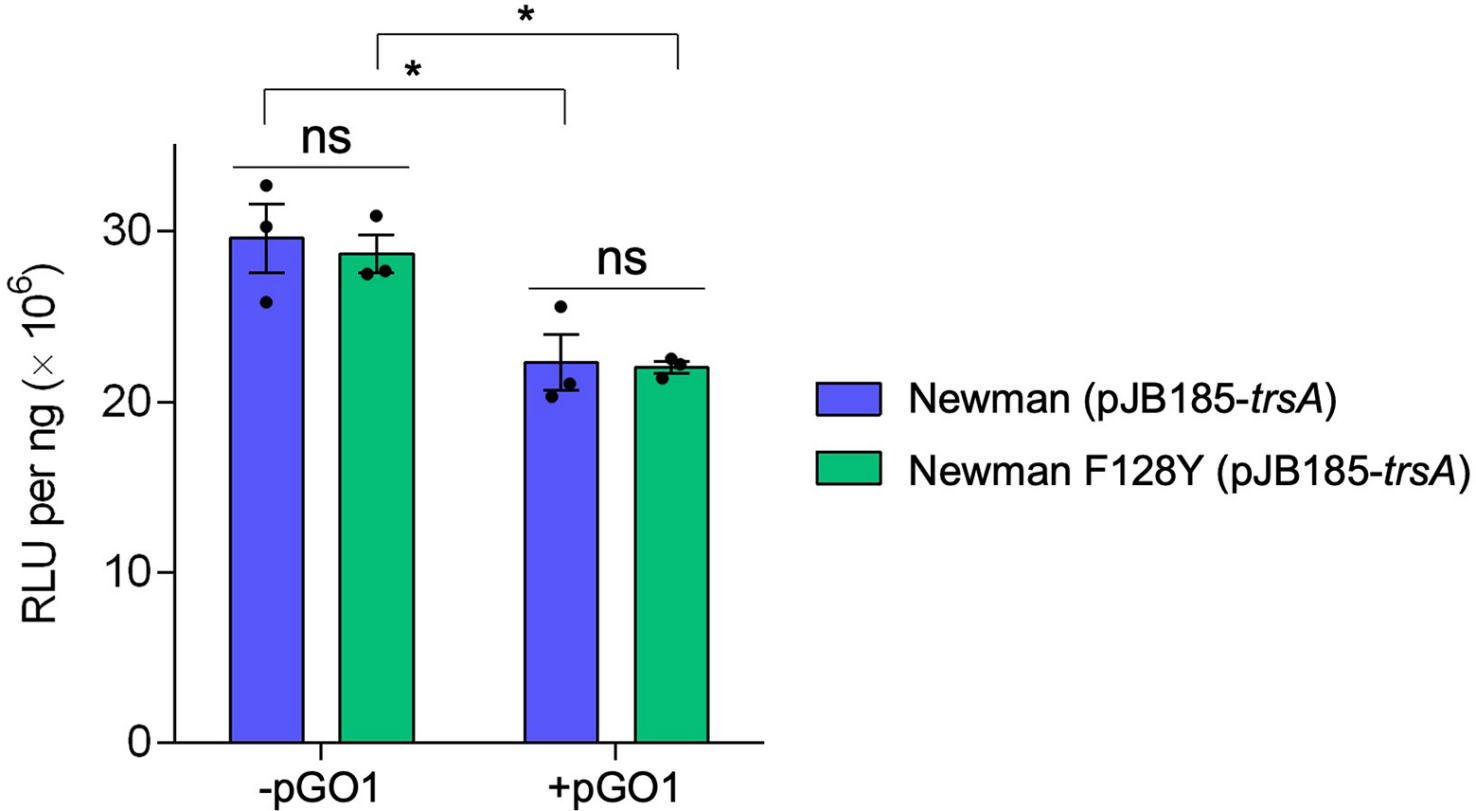
Clinical Rel mutation does not affect the expression of the conjugation machinery gene *trsA*. LacZ activity in different strains carrying pJB185-*trsA* (a reporter construct in which expression of *lacZ* is under the control of the pGO1 *trsA* promoter) in the presence/absence of pGO1. Data were corrected for total protein content and are derived from three independent replicate cultures per strain (and two technical replicates). Error bars represent the sem. Asterisks indicate statistically significant differences between means as determined by a one-way ANOVA with Tukey’s multiple comparisons test (*P≤0.05; ns=not significant).

Our data indicate that pGO1 PCN is not altered in our F128Y mutant. While increased PCN can lead to increased conjugation frequency via transfer gene dosage effects [11], transfer gene expression can also be modulated directly by its repressor [19]. Given that (p)ppGpp is known to bind to many diverse proteins with no specific binding motif [36], we hypothesized that (p)ppGpp could be binding to the pGO1 repressor TrsN and relieving its repression of transfer genes. TrsN binds to several transfer gene promoters, including that of *trsA* [19]. Therefore, we constructed a *trsA* expression reporter, where the *trsA* promoter drives expression of LacZ. When this construct was introduced into Newman wild-type and the F128Y mutant with and without pGO1, the repressive effect of TrsN expressed from pGO1 on the expression of *lacZ* was clear (Fig. 6). However, there was no significant difference in LacZ activity between the wild-type and mutant, indicating no difference in *trsA* expression.

To more widely explore the differences between wild-type Newman and F128Y that could impact conjugation frequency, we performed RNA sequencing on each strain bearing pGO1 and grown under conjugation assay conditions (i.e. on filters but in the absence of recipient). The ability and sensitivity of this bulk sequencing approach to detect any differences in expression of plasmid-encoded genes is severely limited as conjugation is a rare event and typically only a small fraction of the donor population is conjugation-competent at any one time [72]. Furthermore, the presence of recipient is known to induce plasmid gene expression in other bacterial species [72]. Nevertheless, we compared the plasmid transcriptomes of the two strains with these limitations in mind. Of the 57 transcripts originating from pGO1, one was differentially expressed (log2 fold change >1 [equal to >twofold change] and adjusted *P*-value<0.05), with 3.5-fold greater expression in wild-type Newman: MobV family relaxase (locus tag PGO1_p19). Plasmid-encoded relaxases are involved in cleaving plasmid DNA at the *oriT* and initiating conjugative transfer; however, PGO1_p19 is not the canonical and well-studied pGO1/pSK41 relaxase, NES, that is encoded next to *oriT* and considered to be part of the core pGO1 conjugative replicon [73,74]. Furthermore, downregulation of a relaxase in the F128Y mutant does not intuitively align with *increased* conjugation frequency. If the threshold for differential expression is lowered to a 1.5-fold change, expression of NES was also higher in the wild-type than the mutant (as well as two neighbouring genes encoding for small hypothetical proteins on the opposite strand). With this lower fold-change threshold, two genes were more highly expressed in the mutant than the wild-type: a putative resolvase, known as Res [75], and a 55 amino acid hypothetical protein. Plasmid-encoded resolvases act to resolve plasmid dimers into monomers during partitioning and contribute to plasmid maintenance [76]. We could find no precedent for a relationship between resolvase activity and conjugation frequency in the literature. However, overlap between tolerance mutations and increased plasmid maintenance has been reported in *E. coli* [77]. Manual inspection of all reads that mapped to pGO1 – including antisense and intergenic regions – revealed no other differences between the two datasets.

Moving on to chromosomal gene expression, 96 chromosomal genes were upregulated in the F128Y mutant and 163 genes were downregulated (>twofold change and adjusted *P*-value<0.05). The most striking differential expression was the upregulation of capsule biosynthesis genes in the F128Y mutant (2.5–3.5-fold change). This same property was observed in the original clinical isolate bearing the F128Y mutation via microarray analysis [78] and these genes are part of the CodY regulon [79]. Capsule type and volume have been shown to impact conjugation efficiency in Gram-negative bacteria (where capsule volume is negatively correlated with conjugation efficiency) [80]. However, we observed elevated conjugation frequency in a *rel* F128Y mutant of USA300 LAC (Fig. S2), which lacks a capsule [81]. Therefore, the upregulation of capsule biosynthesis genes in the F128Y mutant is unlikely to be the cause of elevated conjugation frequency.

More generally, of the 145 CodY regulon genes identified in our datasets [82], only 20 were upregulated in the F128Y mutant compared with wild-type (12 being capsule biosynthesis genes). The total number of differentially expressed genes in F128Y (259 genes) is small compared with the 1074 genes/sRNAs affected when the stringent response is induced via induction of the Rel synthetase domain in a (p)ppGpp-null background [83]. Instead, the number of genes affected is more similar to that detected upon *relQ* induction (152 genes/sRNAs), which represents very low-level stringent response induction [83]. Overall, the transcriptome of the F128Y mutant contains some (but not all) of the hallmarks of elevated (p)ppGpp, with upregulated purine synthesis and downregulation of many catabolic processes (Fig. S4) [82]. Among these differentially expressed genes, none were noted to have previously been linked to conjugation frequency (e.g. biofilm formation, cell membrane composition and cell permeability [22,56, 84]).

## Discussion

The stringent response is a master regulator of many bacterial physiological processes – from growth, metabolism and sporulation to biofilm formation, virulence expression and antibiotic tolerance [38,39]. The results presented here suggest that plasmid conjugation should be added to this list. Using two mutant strains that carry clinical Rel mutations, as well as chemical and further genetic modulation of the stringent response, we have shown that elevated (p)ppGpp levels lead to higher rates of plasmid donation. This effect applies to plasmids from the three different families of staphylococcal plasmid, in addition to a mobilizable plasmid. Experimental investigations into potential plasmid-mediated factors revealed none of the typically reported properties observed in strains with high conjugation frequency (i.e. increased PCN or upregulated conjugation machinery expression). While we did observe an increase in gentamicin resistance in the F128Y mutant compared with wild-type, we hypothesize this may be due to reduced uptake of the aminoglycoside (common among small colony variants [81]), rather than increased PCN. Our transcriptomic analysis of pGO1 is greatly limited by the absence of recipient; however, it did reveal some small changes in a limited number of genes. While none of these genes have a known association with conjugation frequency, they may still warrant further investigation. It is important to note that elevated (p)ppGpp typically leads to a global downregulation of catabolic processes, including RNA synthesis; therefore, the ~1.5 fold upregulation of two plasmid-encoded genes in the F128Y mutant may be more significant than they appear.

Host factors can also impact conjugation frequency and must be considered here. An obvious difference between our Rel mutants and the wild-type is lag time and/or growth rate [42]. However, higher rates of plasmid transfer are typically associated with *faster* growing bacteria [85,86] (although there are examples of plasmids that transfer at higher frequencies from stationary-phase donors than exponentially growing donors [87]). It is interesting and intriguing that elevated (p)ppGpp affects plasmid donation, but not plasmid uptake. Both plasmid transfer (by the donor) and acquisition (by the recipient) are thought to impose a significant metabolic burden [88]. Plasmid acquisition results in metabolic costs associated with the expression of additional genes and proteins, while plasmid transfer is an ATP-costly process due to the necessary expression of the conjugation machinery [88]. While antibiotic-tolerant bacteria often have lower ATP levels than their non-tolerant counterparts [89], these slow-growing bacteria are using less energy overall for chromosomal replication, protein synthesis and general growth; therefore, perhaps Rel mutant donors have more resources available to direct towards conjugation. Stressed bacteria are also known to exhibit high conjugation frequencies (although the effect varies with stressor and concentration) [22,24, 29, 30, 90]. This is thought to potentially be part of a ‘bet-hedging’ strategy on the part of the donor, which could also apply in the case of our Rel mutants. Interestingly, an *E. coli* plasmid-encoded protein, TraR, has been shown to activate an alternative sigma factor and mimic the effects of stringent response activation in the absence of elevated (p)ppGpp, leading to greater tolerance to the stress associated with plasmid transfer [27]. This implies that stringent response activation may be advantageous in a donor. Overall, it is clear that the physiological state of donors and recipients varies and (p)ppGpp levels appear to exclusively impact the donor with regards to conjugation.

Outside of the energetics of plasmid conjugation, many other host factors have been linked to differences in plasmid donation, including cell permeability, cell envelope changes and a range of metabolism-associated genes (albeit largely in Gram-negatives) [21,22, 84, 91]. Van Wonterghem *et al*. performed a genome-wide association study (GWAS) to look for donor factors that assist conjugative plasmid transfer in *E. coli* [21]. This identified changes in genes associated with motility, anabolism and catabolism and molecular/ion transport (potentially affecting intracellular pH and salinity). A high-throughput screen for chromosomal genes controlling F-plasmid donation in *E. coli* also identified >50 novel genes whose deletion led to defective plasmid donation [91]. Among these genes, overrepresented cellular functions included DNA replication, protein folding and lipopolysaccharide (LPS) synthesis. The bacterial cell envelope, including LPS in Gram-negatives, represents a physical barrier to plasmid transfer that is spanned by the mating pore. As such, changes to the cell envelope structure have been shown to affect conjugation frequency in *E. coli* and *Bacillus subtilis* [84,92]. In *E. coli*, exposure to subinhibitory concentrations of the membrane-active antibiotic polymyxin B reduced membrane permeability and conjugation frequency, while high concentrations increased membrane permeability and conjugation frequency concomitantly [92]. However, other concurrent changes in stress responses and cell energetics were also detected, so it is unclear which effect(s) directly impacted plasmid donation rate. Finally (and perhaps most relevant to our observations with *S. aureus*), changes in the phospholipid composition of the cytoplasmic membrane of *B. subtilis* have been shown to affect the conjugation efficiency of an integrative and conjugative element [84,93]. A loss of lysyl-phosphatidylglycerol from the membrane brought about by an *mprF* deletion led to a decrease in conjugation, while overexpression of *mprF* led to an increase in this phospholipid and conjugation frequency. However, unlike our observations with Rel mutant strains, this effect was observed when either the donor or recipient was an *mprF* mutant. Other differences in conjugation frequency were observed in phospholipid synthesis-associated gene deletion mutants independent of lysyl-phosphatidylglycerol content; therefore, more general changes in phospholipid synthesis and membrane composition may impact conjugation frequency.

While we were unable to match any of these reported host factor associations with our transcriptomic analysis, they warrant further investigation as possible targets of (p)ppGpp regulation and explanations for our findings. At its peak, (p)ppGpp can regulate expression of up to a quarter of the genome [94]. (p)ppGpp also has significant post-translational effects, binding to a range of proteins and enzymes, modifying their activity [36]. Among these proteins are those involved in: transcription; translation; amine, amino acid, nucleotide, carbon and fatty acid metabolism; DNA replication; and second messenger modulation [36]. Therefore, (p)ppGpp could be exerting an effect on conjugation in our Rel mutant donors by interacting with one or more plasmid- or chromosomally-encoded proteins. Overall, further work is required to determine the molecular basis of increased plasmid donation associated with elevated (p)ppGpp. This could include live-cell imaging of the dynamics of different stages during the conjugation process [95], measurement of cell permeability and membrane lipid composition and (p)ppGpp pulldowns of plasmid-carrying cells [96,97]. The implications of this newly identified relationship between (p)ppGpp and conjugation in *S. aureus* are significant. Mutations in Rel and accessory (p)ppGpp synthetases are increasingly being identified among clinical isolates [46,48,78], where they already confer antibiotic tolerance and impact the expression of resistance [42,43]. Our findings suggest that these mutations can also promote the dissemination of resistance plasmids, contributing to the wider problem of resistance. Due to the role of the stringent response in bacterial growth, stress survival and virulence, it is already under investigation as a target for novel antibiotics [98]. The emerging relationship between (p)ppGpp and plasmid dissemination reported here adds further impetus to this search, with the potential to prevent plasmid dissemination, reverse antibiotic tolerance and reduce virulence with one therapeutic.

## Supplementary material

10.1099/mic.0.001724 Supplementary Material 1.

## Abbreviations

CAT: chloramphenicol acetyltransferase
ddPCR: droplet digital polymerase chain reaction
GO: gene ontology
GWAS: genome-wide association study
LB: Lysogeny Broth
MGE: mobile genetic element
MIC: minimum inhibitory concentration
NOV^R^: novobiocin-resistant
PCN: plasmid copy number
(p)ppGpp: guanosine tetra- and pentaphosphate
TSA: tryptic soy agar
TSB: tryptic soy broth
