# Supplementary material for: Clinical Rel mutations that increase basal (p)ppGpp promote conjugal transfer of staphylococcal resistance plasmids
Source: Microbiology (Reading). 2026 Jun 9;172(6):001724. doi: 10.1099/mic.0.001724 (PMC13249265; doi:10.1099/mic.0.001724)
Supplement: Supplementary Material 1. [file mic-172-01724-s001.pdf]

SUPPLEMENTARY MATERIAL

Clinical Rel mutations that increase basal (p)ppGpp promote conjugal transfer of staphylococcal resistance plasmids

Ashley T. Deventer, Ava Sutherland, Daria Biernacka, Paul R. Johnston, Claire E. Stevens, Anna-Karina Kaczorowska, Alisdair B. Boraston and Joanne K. Hobbs

Table S1. List of bacterial strains and plasmids used in this study

Bacterial strain/plasmid	Description	Source/Reference
**Bacterial strains**		
Newman NOV^R	Novobiocin-resistant version of wildtype Newman bearing *gyrB* R144I mutation	Deventer et al. (43)
Newman *rel* F128Y NOV^R	Novobiocin-resistant version of Newman *rel* mutant bearing *gyrB* R144I mutation	Deventer et al. (43)
Newman *rel* F128Y comp NOV^R	Novobiocin-resistant version of complemented Newman *rel* mutant bearing *gyrB* R144I mutation	This study
Newman *rel* F128Y comp FUS^R	Fusidic acid-resistant version of complement Newman *rel* mutant	This study
Newman *rel* L152F NOV^R	Novobiocin-resistant version of Newman *rel* mutant bearing *gyrB* R144I mutation	Deventer et al. (43)
Newman *rel* L152F comp NOV^R	Novobiocin-resistant version of complemented Newman *rel* mutant bearing *gyrB* R144I mutation	This study
USA300 LAC NOV^R	Novobiocin-resistant version of wildtype USA300 LAC bearing *gyrB* R144I mutation	This study
USA300 LAC *rel* F128Y NOV^R	Novobiocin-resistant version of USA300 LAC *rel* mutant bearing *gyrB* R144I mutation	This study
Newman Δrel_{syn}	Newman mutant bearing Δ924-930 deletion in Rel synthetase domain	This study
Newman Δrel_{syn} NOV^R	Novobiocin-resistant version of Newman Δrel_{syn} bearing *gyrB* R144I mutation	This study
Newman Δrel_{syn} Δ*relP* Δ*relQ*	Newman mutant bearing Δ924-930 deletion in Rel synthetase domain and deletions of *relP* and *relQ*	This study
Newman Δrel_{syn} Δ*relP* Δ*relQ* NOV^R	Novobiocin-resistant version of Newman Δrel_{syn} Δ*relP* Δ*relQ* bearing *gyrB* R144I mutation	This study
Newman Δ*codY*	Newman mutant bearing *codY* deletion	This study
Newman Δ*codY* NOV^R	Novobiocin-resistant version of Newman Δ*codY* bearing *gyrB* R144I mutation	This study

Newman *lexA* S130A	Newman mutant bearing S130A mutation in *lexA*	This study
Newman *lexA* S130A NOV^R	Novobiocin-resistant version of Newman *lexA* S130A bearing *gyrB* R144I mutation	This study
RN4220	Restriction-deficit laboratory strain	Fairweather et al. (99)
SH1000	Standard laboratory strain	Horsburgh et al. (100)
SH1000-NR	Novobiocin- and rifampicin-resistant version of SH1000	This study
WBG541	Fusidic acid- and rifampicin-resistant laboratory strain	Udo et al. (84)
WBG4515	Streptomycin- and novobiocin-resistant laboratory strain	Townsend et al. (87)
**Plasmids**		
pGO1	Prototypical conjugative staphylococcal plasmid. Encodes for gentamicin and trimethoprim resistance. Member of pGO1/pSK41 plasmid family. 54 kb.	Dr Alex O'Neill, University of Leeds<br><br>Caryl & O'Neill (9)
pWBG707	Conjugative staphylococcal plasmid. Encodes for trimethoprim resistance. Member of the pWBG4 plasmid family. 38 kb.	Dr Josh Ramsay, Curtin University<br><br>Udo et al. (86)
pWBG749e	Erythromycin-resistant derivative of conjugative staphylococcal plasmid pWBG749 carrying Tn551. Member of the pWBG749 plasmid family. 43 kb.	Dr Josh Ramsay, Curtin University<br><br>O'Brien et al. (85)
pC221	Mobilisable staphylococcal plasmid. Encodes for chloramphenicol resistance.	Dr Alex O'Neill, University of Leeds<br><br>Projan et al. (88)
pSK5487	Plasmid copy number *cat* reporter construct carrying pGO1/pSK41 replication region.	Dr Stephen Kwong, Western Sydney University<br><br>Kwong et al. (14)
pJB185	*S. aureus lacZ* reporter construct.	Dr Jeffrey Bose, University of Kansas<br><br>Krute et al. (90)

pJB185-*rep*	pGO1 *rep* promoter-*lacZ* fusion construct	This study
pJB185-*trsA*	pGO1 *trsA* promoter region-*lacZ* fusion construct	This study

Table S2. Primers used in this study

**Primer name**	**Use**	**Primer sequence (5'→3')**
rel upstream fwd	Amplification of *rel* gene half	ATAAGCTTGATATCGATGAAC AACGAATATCCATATAGTGC
rel downstream rev	Amplification of *rel* gene half	ACCGCGGTGGCGGCCCTAG TTCCAAACTCTTGTTACTGTATAAAC
rel-syn upstream rev	Amplification of *rel* gene half lacking nucleotides 924-930	TACTGTAGTATGCAACAAATT TTGTTTAGGCATTGCAATATAATC
rel-syn downstream fwd	Amplification of *rel* gene half lacking nucleotides 924-930	CCTAAACAAAATTTGTTGCAT ACTACAGTAGTAGGACC
rel seq fwd	Amplification of *rel* and flanking sequence for sequencing confirmation	AGGAATAGTATACAAATTAAACTCGC
rel seq rev	Amplification of *rel* and flanking sequence for sequencing confirmation	GTAGAGTTCTGACCGATACC
relP upstream fwd	Amplification of *relP* gene half	ATAAGCTTGATATCGATAAGC TTGATATCGTATCAGCCCAAAGTTCGATAGTG
relP downstream rev	Amplification of *relP* gene half	ACCGCGGTGGCGGCCTTA ATTGTTATGTTGTATGTGGGATATTTCTAATTG
relP upstream rev	Amplification of *relP* gene half lacking nucleotides 450-536	CATATCCATACCTATAATATAAT CTTTACGTTTTATCAATTGTACGTCTTC
relP downstream fwd	Amplification of *relP* gene half lacking nucleotides 450-536	CGTAAAGATTATATTATAGGTAT GGATATGTGGGCAAGTTTAG
relP seq fwd	Amplification of *relP* and flanking sequence for sequencing confirmation	ATCGTACTTTGATAGCGAATCAATTGG
relP seq rev	Amplification of *relP* and flanking sequence for sequencing confirmation	TCAAAGTCACTCCTTCATTACACG
relQ upstream fwd	Amplification of *relQ* gene half	ATAAGCTTGATATCGATAAGCTTGATATCGATGC GTTTATATATTAATGAAATTAAAATTAAAGATGAC

relQ downstream rev	Amplification of *relQ* gene half	ACCGCGGTGGCGGCCTCA<br>TCGTTCTTCATCACTTGATATGAAAG
relQ upstream rev	Amplification of *relQ* gene half lacking nucleotides 343-429	GAAATTCATTGCTAAACCACTTTCTTTAGTGTTA<br>CGAATATAATCTC
relQ downstream fwd	Amplification of *relQ* gene half lacking nucleotides 343-429	ACTAAAGAAAGTGGTTTAGCAATGAATTTCTGG<br>GCAAC
relQ seq fwd	Amplification of *relQ* and flanking sequence for sequencing confirmation	TTGCCATGATATGTATACACCTCG
relQ seq rev	Amplification of *relQ* and flanking sequence for sequencing confirmation	AGGTATTAAATTAACACTCGGTATTTCTCG
codY upstream fwd	Amplification of *codY* upstream flanking sequence	ATAAGCTTGATATCGATAAGCTTGATATCGA<br>TGGATACAGCTGGAATAAGATTAACTCC
codY upstream rev	Amplification of *codY* upstream flanking sequence	CATGAATTTTTCTCCTTTTGTATATTTTTATA<br>GAATAAATGC
codY downstream fwd	Amplification of *codY* downstream flanking sequence	GGAGAAAAATTCATGTAAGTCGATGAGTCT<br>GGGACATAATTC
codY downstream rev	Amplification of *codY* downstream flanking sequence	ACCGCGGTGGCGGCCTTATTCTTCAGTTG<br>CTTCTGTTTCTTCTG
codY seq fwd	Amplification of *codY* flanking sequence for sequencing confirmation	TATTGGATCAGGAGGCAACTACG
codY seq rev	Amplification of *codY* flanking sequence for sequencing confirmation	CTGAAATAGTTGCCATTCATTATTCCTCC
lexA upstream fwd	Amplification of *lexA* gene half	ATAAGCTTGATATCGAACGCTATTTCGC<br>AAAAATAGGCAAAATAACG
lexA downstream rev	Amplification of *lexA* gene half	ACCGCGGTGGCGGCCCAGATAAACCAA<br>AAGATGAGGATATACTTGAACGC
lexA downstream fwd	Amplification of *lexA* gene half with S130A mutation	CGTAGGCGACGCTATGATTGAG
lexA upstream rev	Amplification of *lexA* gene half with S130A mutation	AATCATAGCGTCGCCTACGACG
lexA seq fwd	Amplification of *lexA* and flanking sequence for sequencing confirmation	CTCCTTTGCTTCTTCTTGAGTTAATCC

lexA seq rev	Amplification of *lexA* and flanking sequence for sequencing confirmation	TATGCACATCAAGGATTAATGAATTCTATTGG
pJB185 amp fwd	Amplification of pJB185 backbone	TCTAGAATGACAATGATTACAGATTCATTAGC
pJB185 amp fwd	Amplification of pJB185 backbone	AATTCGTAATCATGTCATAGCTGTTTCC
pGO1 *rep* fwd	Amplification of pGO1 *rep* promoter region	ACATGATTACGAATTATATAATTGACCTGTGAGGCAAC
pGO1 *rep* rev	Amplification of pGO1 *rep* promoter region	CATTGTCATTCTAGACATGATAAAAACTCCTTTAAATGTATATTTAAGG
pGO1 *trsA* fwd	Amplification of pGO1 *trsA* promoter	TATGACATGATTACGAATTCTGACAAAAAAGTTAAAAAAGTTTTATAAATTACATGAC
pGO1 *trsA* rev	Amplification of pGO1 *trsA* promoter	TCATTGTCATTCTAGACATTTACAACCCACCTTTTCTATTTGATAAATC
pGO1 *aphD* fwd	Amplification of aphD gene fragment using ddPCR	CAAGAGCAATAAGGGCATACC
pGO1 *aphD* rev	Amplification of aphD gene fragment using ddPCR	TTCATTGCCTTAACATTTGTGGC
femA fwd	Amplification of femA gene fragment using ddPCR	CATGGCATTGACCGTTATAATTTC
femA rev	Amplification of femA gene fragment using ddPCR	CACCAACATATTCAATAATTTCAGCA

Underlined nucleotides are complementary to the vector and used for In-Fusion cloning

SUPPLEMENTARY REFERENCES

99. Fairweather N, Kennedy S, Foster TJ, Kehoe M, Dougan G. 1983. Expression of a cloned *Staphylococcus aureus* alpha-hemolysin determinant in *Bacillus subtilis* and *Staphylococcus aureus*. Infect Immun 41:1112.

100. Horsburgh MJ, Aish JL, White IJ, Shaw L, Lithgow JK, Foster SJ. 2002. sigmaB modulates virulence determinant expression and stress resistance: characterization of a functional *rsbU* strain derived from *Staphylococcus aureus* 8325-4. J Bacteriol 184:5457.

SUPPLEMENTARY FIGURES

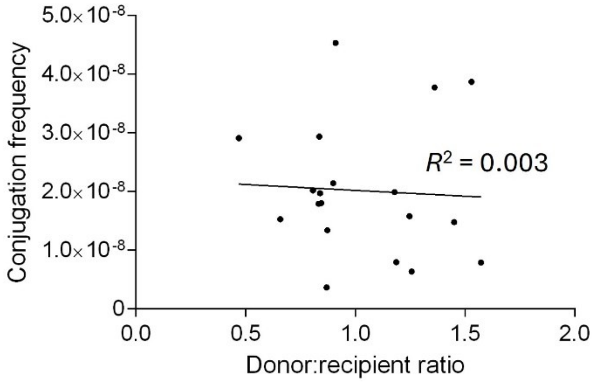

Figure S1. Effect of donor-to-recipient ratio on conjugation frequency. Conjugation frequency and donor-to-recipient ratio data for wildtype Newman (pGO1) matings with SH1000-NR were collated from different experiments/days and plotted. A linear regression line was fitted in GraphPad Prism and the goodness of fit (R^2) is shown.

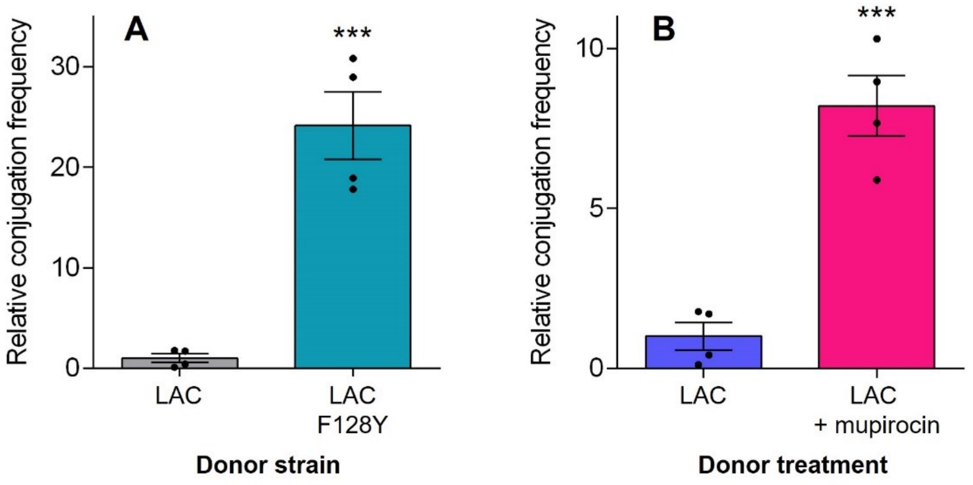

Figure S2. Rel mutation and mupirocin exposure both increase the rate of pGO1 donation in USA300 LAC. Filter matings were performed with the donor indicated carrying pGO1, and SH1000-NR as the recipient. For LAC + mupirocin, the donor was exposed to a subinhibitory concentration of mupirocin prior to mating. All conjugation frequencies were calculated as transconjugants per donor and are expressed relative

to the LAC mean. Data shown are the mean of four biological replicates; errors bars represent the SEM. Asterisks indicate statistically significant differences between means as determined by a two-tailed *t*-test (*** = *P* ≤0.001).

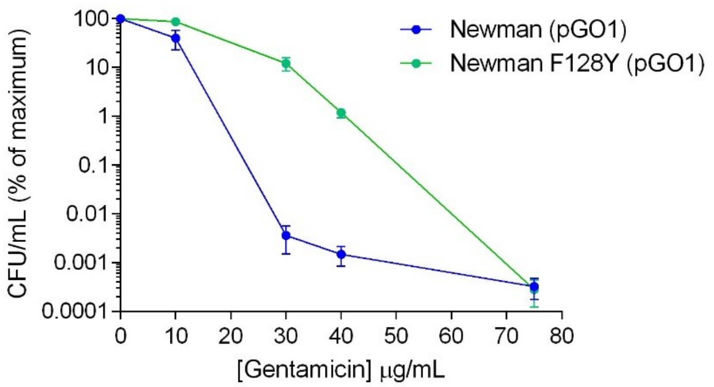

Figure S3. Population analysis profile of wildtype Newman and the Rel F128Y mutant carrying pGO1 with gentamicin. Triplicate overnight cultures were diluted, plated on TSA containing increasing concentrations of gentamicin, and the number of colonies counted following incubation. For each replicate culture, the number of CFU/mL at each gentamicin concentration were expressed as a percentage of the maximum CFU/mL determined in the presence of no gentamicin. Error bars represent the SEM.

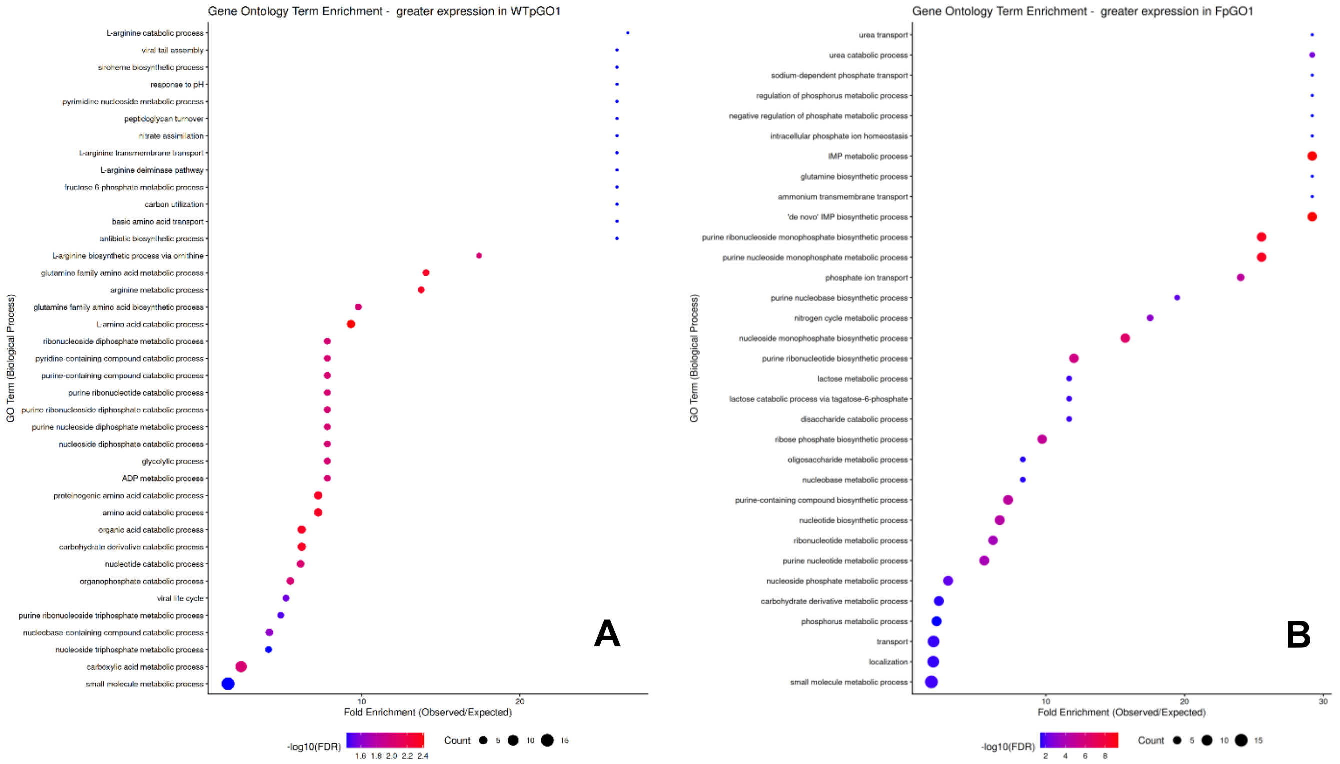

Figure S4. Gene ontology (GO) term enrichment plots for differential expression between F128Y (pGO1) and Newman (pGO1). (A) GO term enrichment analysis for all genes upregulated in Newman (pGO1) *vs* F128Y (pGO1). (B) GO term enrichment analysis for all genes upregulated in F128Y (pGO1) *vs* Newman (pGO1). Differential gene expression was determined by a threshold of absolute fold change greater than 2 and an FDR-adjusted *p* value less than 0.05 with four biological replicates.
